# Within- and Between-Subject Analyses of the Effects of Chronic Xylazine on Negative Phototaxis in Two Planarian Species

**DOI:** 10.3390/biom15111542

**Published:** 2025-11-02

**Authors:** Tom Byrne

**Affiliations:** Department of Psychology, Massachusetts College of Liberal Arts, North Adams, MA 01247, USA; t.byrne@mcla.edu

**Keywords:** xylazine, chronic administration, single-case experimental design, reversal design, multiple-baseline design, planarians, negative phototaxis

## Abstract

Xylazine, an adulterant found frequently in illicit fentanyl, has been implicated in causing several adverse effects in human recreational users, including skin lesions and complications in the treatment of opiate overdose. Despite these public health concerns, the literature on the basic behavioral effects of xylazine is limited. Recent research has demonstrated that planarians show potential as an emerging and practical animal model for studying the behavioral effects of acute xylazine exposure. The goal of the current investigation was to evaluate the behavioral effects of chronic xylazine administration on negative phototaxis in two planarian species: *Girardia tigrina* and *Schmidtea mediterranea*. Three experiments were conducted. Overall, 10 µM of chronic xylazine exposure, arranged according to a multiple-baseline design, impaired negative phototaxis in *S. mediterranea* but not *G. tigrina*. An ABA reversal design indicated that behavioral effects in *S. mediterranea* abated when chronic xylazine was terminated. Finally, a between-group design replicated potential interspecies differences when *G. tigrina* and *S. mediterranea* were compared directly, with the latter showing significantly greater susceptibility to drug effects. This work provides evidence of the utility of a planarian model for studying the behavioral effects of xylazine and lays the foundation for further investigation into the chronic effects of the drug.

## 1. Introduction

In recent years, xylazine, an adrenergic agonist which also has affinity for kappa opioid receptors [1] traditionally used as an anesthetic in veterinary medicine, has been detected with increasing frequency in illicit supplies of fentanyl [2,3,4,5]. This is cause for concern, as xylazine has been implicated in several adverse health effects, including the development of serious skin lesions [6,7,8], complicating treatment for opiate overdose [9], producing respiratory and cardiovascular impairments [10,11] and potentiating opiate withdrawal [12]. Whether recreational users purposively seek out xylazine is unclear, but some surveys suggest that users do not expect to receive xylazine when purchasing illicit fentanyl [13] or stimulants [14].

Outside of its use as a veterinary surgical anesthetic, preclinical research on the behavioral effects of xylazine is sparse. Perhaps this is because, with the exception of some specific geographic regions, most notably Puerto Rico [15], widespread recreational use is relatively recent. There is essentially no literature base on the drug’s effects on learning and memory. Recent research has shown that xylazine fails to function as a reinforcer itself, nor does it enhance fentanyl reinforcement under animal drug self-administration and conditioned place preference (CPP) assays. Khatri et al. [16] demonstrated that xylazine, whether delivered independently by an experimenter or included in response-dependent intravenous infusions, suppressed responding for fentanyl in both male and female rats. Similarly, Acosta-Mares et al. [17] found that xylazine did not engender CPP in laboratory mice, nor did it potentiate the rewarding effects of fentanyl under the procedure. This makes the rationale for its inclusion as an adulterant in fentanyl somewhat of a scientific mystery. However, Bender et al. [18] demonstrated that xylazine may potentiate the discriminative stimulus effects of fentanyl in laboratory rats, indicating the possibility that xylazine could enhance the subjective effects of fentanyl in humans.

During an era of significant change in government policies for research funding [19], there is an impetus for the identification of cost-effective animal models for studying the behavioral effects of drugs. Invertebrate models may be an enticing option, as they do not require the same level of cost and regulatory oversight as vertebrates. In the United States, invertebrates (with the exception of cephalopods) are exempt from review by most Institutional Animal Care and Use Committees (IACUCs). Although it behooves researchers to conduct their work ethically in terms of both experimental protocols and husbandry procedures, invertebrate research does not require the cost of consulting veterinarians or laboratory inspections. In this regard, planarians, small aquatic flatworms, have considerable promise for assessing drug effects. They possess many of the same neurotransmitters as vertebrates [20,21] and pharmacological investigations using planarians have demonstrated orderly behavioral effects of numerous psychoactive substances, including amphetamine [22], caffeine [23,24], cocaine [25,26], ethanol [27,28], fluoxetine [29,30], histamine [31] opioids [32,33], and nicotine [34,35].

Recently, Taylor et al. [36] examined the effects of xylazine on negative phototaxis in the planarian species *Girardia tigrina*. Negative phototaxis is an innate tendency in planarians to move away from light [37]. Xylazine produced orderly effects on the speed of negative phototaxis, impairing the behavior in a dose-dependent fashion, demonstrating that planarians are susceptible to the behavioral effects of the drug. Larger doses first elicited stereotypy (the planarians engaged in C-shaped and corkscrew hyperkinesia), which coincided with the absence of negative phototaxis. This was followed by a period of sedation with little to no motor activity. In addition, the authors demonstrated that physical stimulation reversed the sedative effects of xylazine in several subjects. They utilized a multiple-baseline design in which the time of physical stimulation was staggered across individual planarians so that the manipulation would not be confounded with pharmacokinetics.

Multiple-baseline designs are a type of single-case experimental design (SCED) which involve exposing each subject in an experiment to all control and experimental conditions [38]. SCEDs may have several strengths for investigating the behavioral effects of drugs in planarians. First, they allow for precise documentation of variability in drug responsiveness among individual subjects; the texture of which may be buried if investigators report group means only. Second, these designs include built-in replications [39,40] A single experiment provides numerous opportunities to determine whether behavior changes occur at the time of drug administration. Finally, because relatively few subjects are needed compared to between-group designs, they exemplify the concept of reduction [41], a widely accepted ethical principle that promotes minimizing distress in animal research by limiting exposure to potentially aversive manipulations to no more than what is scientifically necessary.

One limitation to the translational value of the work by Taylor et al. is that those authors investigated only acute administration. Preference for fentanyl as a drug of choice among individual opiate users is correlated with higher rates of drug use [42]. Therefore, it is likely that those who administer xylazine, either knowingly or unknowingly, may do so chronically, resulting in measurable body burdens of xylazine persisting between administrations. Chronic administration may produce several pharmacological phenomena, including tolerance, sensitization, and physical dependence. Thus, it may be fruitful to further assess the utility of planarians as an animal model by investigating the effects of repeated xylazine administration.

The current investigation had three main goals. The first was to extend the work of Taylor et al. by examining the chronic effects of xylazine on negative phototaxis in planarians. The second goal was to assess the viability of *Schmidtea mediterranea*, in addition to the *G. tigrina* species used by Taylor et al., as a potential animal model for investigating the behavioral effects of xylazine. Although there is a growing body of research using planarians in pharmacology studies, no definitive guidelines exist for species selection, and it is quite possible that different species will respond differentially to drug effects. As Martinez et al. [43] noted, “Even if different species of planarians look morphologically similar, they might behave differently when exposed to the same stimuli” (p. 1). *S. mediterranea* is a promising and popular model for genetics and tissue regeneration research [44,45], and documenting effects of xylazine in both species may enhance the generality of findings. I am unaware of any direct cross-species comparisons of the behavioral effects of drugs in *G. tigrina* and *S. mediterranea*. The final goal was to further evaluate the utility of single-case experimental designs for planarian pharmacology by testing both multiple-baseline and reversal designs. Reversal designs comprise at least three phases. First, there is a baseline in which behavior is measured in the absence of manipulations of the independent variable. Second, there is a treatment phase in which the independent variable is applied. Finally, there is a reversal phase in which the independent variable is withdrawn, and behavior is again recorded under baseline conditions.

## 2. General Materials and Methods

### 2.1. Subjects

*Girardia tigrina* were acquired initially from Carolina Biological Supply (Burlington, NC, USA). *Schmidtea mediterranea* were provided by the Stowers Institute for Medical Research (Kansas City, MO, USA). The two species were group-housed separately in Montjuïc water (1.6 mM NaCl, 1.0 mM CaCl_2_, 1.0 mM MgSO_4_, 0.1 mM MgCl_2_, 0.1 mM KCl, 1.2 mM NaHCO_3_) for several months prior to the commencement of experimental procedures. At the start of the experiments, individual planarians approximately 1.5 cm in length were removed from the colonies and housed individually in Petri dishes containing 15 mL of Montjuïc water. All planarians were kept in the dark at an ambient temperature of approximately 23 °C. They were fed beef liver once per week, and initial experimental sessions were conducted approximately 48 h post-feeding for each planarian. No further feeding occurred once trials began for a given planarian. Because planarians are invertebrates, our procedures were exempt from Institutional Animal Care and Use Committee (IACUC) review.

### 2.2. Apparatus

Plastic Petri dishes (10 cm diameter) were used for all sessions. A 7-wattt LED lamp (Model HD1504C; GLFERA, Xinxiang, China. The manufacturer lists no corporate city on packaging or online product documentation; and the location is reported based on trademark registration records.) was positioned 64 cm above the center of each Petri dish. Light intensity measured at the level of the Petri dish was 352 lux. Black paper was affixed to each Petri dish lid, bisecting it into light and dark sections. An iPad (Apple, Cupertino, CA, USA) mounted above the dish was used for video recording. Temperature readings taken with an infrared thermometer (model 774; Etekcity, Anaheim, CA, USA) at 5 min intervals for 30 min indicated that the lamp had no effect on the temperature of either the opaque or translucent side of the Petri dishes. Planarians were transferred between dishes using 1 mL plastic pipettes.

### 2.3. Drug

Xylazine hydrochloride (Sigma Chemical Co., St. Louis, MO, USA) was dissolved in Montjuïc water to produce a 10 µM solution. Pilot data suggested that extended exposure to this dose would affect planarian behavior without causing mortality. The solution was prepared on the first day of data collection and stored in the dark at an ambient temperature of approximately 23 °C for the duration of data collection, which was approximately 24 days.

### 2.4. General Behavioral Procedures

To record negative phototaxis times, individual planarians were removed from their home Petri dish with a transfer pipette and repositioned against the dish wall. The bisected cover was replaced so that the planarian was approximately equidistant from the two endpoints of the opaque border where it contacted the Petri dish wall. In other words, a line drawn from the planarian to the cover would be perpendicular to the line formed by the black paper. Trials ended when the planarian crossed into the dark half of the Petri dish. Following each trial, planarians were immediately returned to a dark environment. All sessions were video-recorded. The dependent variable, travel time, was obtained by stopping the video at the exact point of pipette expulsion and subtracting that time from the last recorded moment when the planarian was visible.

### 2.5. Data Anaylsis

Prism version 6.0 (GraphPad, San Diego, CA, USA) was used to conduct statistical analyses and generate figures. All statistical analyses were conducted using an alpha level of 0.05. The Percentage of Non-overlapping Data (PND) was calculated by dividing the number of values in the chronic-administration phase that were greater than the highest value recorded during the corresponding baseline phase by the total number of values in the chronic administration phase and then multiplying by 100.

### 2.6. Interobserver Agreement

Phototaxis times were obtained from the video recordings of each trial. Theoretically, this resulted in greater accuracy than scoring the trials in real time, although doing so is common practice in planarian pharmacology research, as the video recordings could be slowed down and stopped for judging the start and end of each trial. However, this still required decisions from human observers. In some cases, reflections and shadows along the wall of the Petri dish may have impaired accuracy, especially when judging the moment when the very end of the planarian’s tail crossed the midline. To assess reliability, 25 trials from across the three experiments were selected, at random, and phototaxis times were scored by a second observer who was blind to experimental conditions (the observer was not informed of the methods, conditions, or experimental questions). Trials in which a planarian failed to cross into the dark half of the Petri dish were excluded from this analysis and replaced because session durations of 600 s, for which there was always perfect agreement between two observers, would have had a disproportionate impact on improving the reliability calculations. To calculate interobserver agreement, the shortest time recorded for each trial was subtracted from the longest time recorded for each trial. The mean difference between observers across the 25 trials was 1.040, with a standard deviation of 1.306.

## 3. Experiment 1

The effects of chronic xylazine on negative phototaxis were tested according to a multiple-baseline design in which each individual planarian was assigned to a different number of baseline trials. This design ensured that potential drug effects would be separated in time from history or maturation threats to internal validity. Such threats may include the amount of experimenter handling, time since last meal, and the possible commencement of reproductive fission, the process by which planarians split into two separate individuals.

### 3.1. Method

Four *G. tigrina* and four *S. mediterranea* were randomly assigned to either two, three, four, or five days of baseline. During baseline sessions, the planarians were housed in the dark in their individual Petri dishes. One phototaxis trial was conducted each day, and trials were separated by 24 h. Following baseline sessions, the Montjuïc water in the planarians’ Petri dishes was replaced with the 10 µM xylazine solution. Twenty-four hours after the start of chronic xylazine exposure, the experimental phase began and continued for five consecutive days for each planarian, with trials conducted once per day at 24 h intervals. The xylazine solution was replaced after each trial.

### 3.2. Results and Discussion

**Figure 1:**
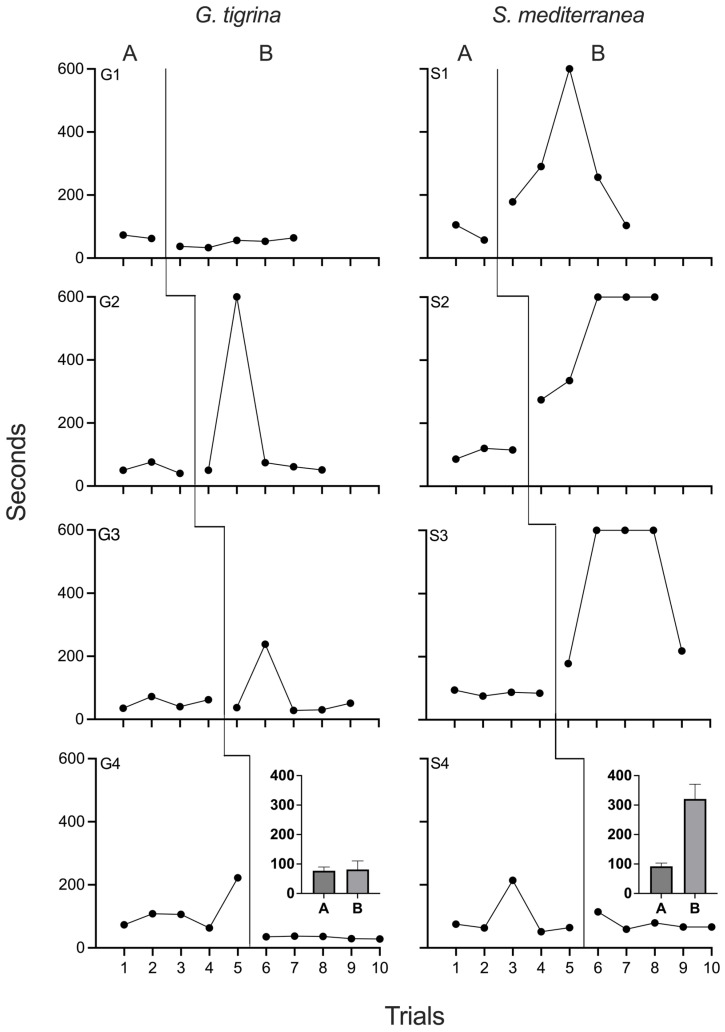
Negative phototaxis times for all *G. tigrina* (left column) and *S. mediterranea* (right column) in Experiment 1. Phase A depicts baseline (no drug) conditions, and Phase B depicts chronic xylazine exposure. The insets in the bottom row depict group means for each phase. Error bars represent 1 SEM.

Figure 1 depicts negative phototaxis data of all planarians under both baseline and chronic xylazine conditions. Baseline travel times for all *G. tigrina* subjects were stable, with no noticeable trends. Travel time during the last baseline session for G4 was an outlier, but this did not affect interpretation, as behavior returned to previous levels during the first day of xylazine exposure. Overall, no consistent effects of chronic xylazine exposure occurred in *G. tigrina*. Travel times for G1 and G4 under chronic xylazine exposure were equivalent to baseline performance, with no changes in travel time or trajectory observed. For G2 and G3, travel times increased significantly during the second trial following chronic exposure but returned to baseline-equivalent levels during subsequent sessions. PND was 0% for G1 and G4 and 20% for G2 and G3.

Stable baseline travel times were also observed for all *S. mediterranea* subjects. However, unlike in *G. tigrina*, chronic xylazine increased travel times in three of the four planarians. Longer times were a function of both slower movement and pausing. These increases occurred during the first session of the chronic xylazine phase, indicating that it was drug exposure and not the number of baseline sessions, which were staggered across individuals, that affected negative phototaxis. For S1, S2, and S3, all travel times were longer than baseline throughout the chronic xylazine phase, and negative phototaxis failed to occur within the 600 s time limit during one session for S1 and three sessions for both S2 and S3. For S1 and S3, travel times decreased again at the end of the phase, but remained slower than baseline levels. No effects of chronic xylazine on negative phototaxis occurred in S4. PND was 80% for S1, 100% for both S2 and S3, and 0% for S4. The absence of drug effects in S4 may indicate individual differences in sensitivity to xylazine. Pronounced individual differences in drug effects have been documented in vertebrates [46] as well as in planarians [27]. The extent of individual variability in the behavioral effects of chronic xylazine in planarians can be determined only with further replication.

The results of Experiment 1 expand upon the work of Taylor et al. [36] by demonstrating that planarians may serve as useful animal models for studying the behavioral effects of xylazine. In both species, baseline measures were reasonably stable, suggesting that single-case experimental designs have potential for measuring drug effects in both species. The multiple-baseline design allowed potential confounders to be separated from exposure to the independent variable. With the exception of one subject, exposure to a chronic dose of 10 µM impaired negative phototaxis in *S. mediterranea* but not *G. tigrina*. This marked difference between the two species was unexpected, and the results should be interpreted with caution, as an explicit between-group comparison was not the goal of the experiment. Instead, Experiment 1 may best be conceptualized as a single experiment in *G. tigrina* that failed to replicate in *S. mediterranea*. The sample size was too small for formal statistical analysis. Nonetheless, examining the effects of chronic xylazine in both species provides a foundation for further investigation.

## 4. Experiment 2

Replication of the findings from Experiment 1, along with testing the utility of an ABA reversal design, were the goals of Experiment 2. Reversal designs are commonly employed in single-case experimental research. The potential value of an ABA design here is twofold. First, it would allow replication of results from Experiment 1 by examining whether negative phototaxis speeds in individuals change at the onset of chronic xylazine exposure and not at other times, thereby bolstering confidence in the existence of a functional relationship. More importantly, reversing experimental conditions by terminating the chronic administration of xylazine would allow for testing of the reversibility of drug effects. If negative phototaxis speeds recover quickly, this would provide evidence that the decrease was due to the behavioral effects of the drug rather than drug-induced toxicity.

### 4.1. Method

Four *G. tigrina* and four *S. mediterranea* were selected from their respective colonies. The conditions for the first three trials were equivalent to the baseline sessions described above for the multiple-baseline design. Following these baseline trials, the Montjuïc water was replaced with a 10 µM xylazine solution, and negative phototaxis trials were conducted at 24, 48, and 72 h of chronic exposure. After the third trial under chronic exposure, the planarians were placed in new Petri dishes containing Montjuïc water. Three additional trials were then conducted at 24 h intervals. An additional trial was allocated to chronic exposure for planarians S7 and S8 due to a decrease in phototaxic time during the third day of exposure. This additional trial was arranged to test if this was an outlier or possibly the start of a trend.

### 4.2. Results and Discussion

**Figure 2:**
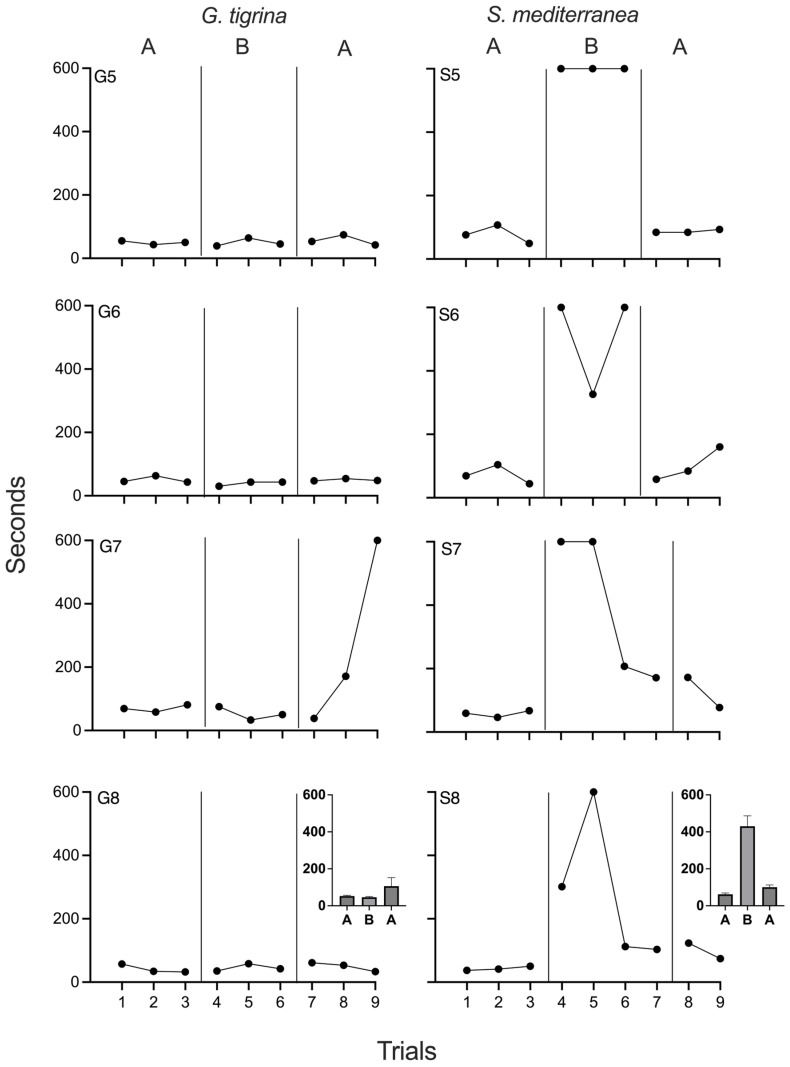
Negative phototaxis times for all *G. tigrina* (left column) and *S. mediterranea* (right column) in Experiment 2. Phase A depicts baseline (no drug) conditions, and Phase B depicts chronic xylazine exposure. The insets in the bottom depict group means for each phase. Error bars represent 1 SEM.

Figure 2 depicts negative phototaxis times for all planarians in Experiment 2. Travel times were stable for both *G. tigrina* and *S. mediterranea* subjects during the first baseline phase. No effects of chronic xylazine were observed in *G. tigrina*, as performance during baseline and drug phases was indistinguishable. For G5, G6, and G8, travel times also remained constant throughout the reversal phase. Travel times for G7 slowed significantly during the final two sessions of the reversal phase. The reasons for the impaired performance are unknown, but were likely due to factors outside of experimental arrangement. When comparing values obtained during chronic xylazine exposure to the first baseline phase, PND was 33% for G5 and G6, and 0% for G6 and G7. PND was 0% for all four *G. tigrina* when comparing times during xylazine exposure to those obtained during the second baseline. *S. mediterranea.*, travel times increased immediately and noticeably at the onset of chronic xylazine exposure. For each of these planarians, all travel times during the drug phase were longer than those recorded during baseline, and all subjects had at least one session of phototaxis failure. S7 and S8 showed decreased travel times during the last two sessions of the drug phase. During the reversal phase, travel times decreased for all *S. mediterranea* and approximated baseline levels, although the reduction in travel times at the end of the chronic xylazine phase for S7 and S8 complicated interpretation of the reversal data for these two individuals. When comparing times obtained during chronic xylazine exposure to the first baseline phase, PND was 100% for all *S. mediterranea*. PND was 100% for S5 and S6, 75% for S7, and 50% for S8 when comparing times during xylazine exposure to those obtained during the second baseline.

Experiment 2 largely replicated the main findings of Experiment 1 by demonstrating that chronic exposure to 10 µM xylazine impaired negative phototaxis in three of four *S. mediterranea* subjects but had no identifiable effects in *G. tigrina*. The utility of a reversal was also evident in two of the *S. mediterranea* subjects, strengthening confidence that it was xylazine exposure, and not the number of sessions or uncontrolled history variables, that led to decreases in negative phototaxis performance. Unlike Experiment 1, no *G. tigrina* subject emitted travel times during drug exposure that were clearly outside the baseline range. This may indicate that the two spikes observed in Experiment 1 were due to variability in negative phototaxis times rather than drug effects.

Overall, the ABA reversal design proved effective for evaluating the behavioral effects of chronic xylazine in planarians. Three baseline sessions, as used here, are considered by some investigators to be the minimum necessary for single-case experimental [47]. Although a greater number of baseline trials would theoretically have more predictive value, baselines in all planarians tested here showed no trends, with phototaxis times occurring in a relatively narrow range. Level changes were evident from the first session of xylazine exposure in all *S. mediterranea*. Even though a downward trend occurred during exposure for two subjects, all data points were outside of the values recorded during baseline. Therefore, conclusions can be drawn that a drug effect was present.

The gradual increase in negative phototaxis speed toward the end of chronic exposure in two of the *S. mediterranea* may be indicative of tolerance, but this hypothesis is speculative and requires replication. The amount of data suggestive of tolerance in the current study must be considered anecdotal and merely suggestive of the possibility. Although chronic drug exposure has rarely been studied in planarians, tolerance has been demonstrated with both morphine [32] and nicotine [35]. A dedicated investigation into the possibility of tolerance would require longer xylazine exposure. Regardless, the return to baseline performance during the reversal phase suggests that the impairment of phototaxis during chronic xylazine exposure was due to a drug effect rather than drug-induced toxicity.

## 5. Experiment 3

Following the unexpected differences in the effects of chronic xylazine between the two species, a third experiment was conducted to compare them directly. Single-case experimental designs (SCEDs) allow for precise analysis of drug effects in individual subjects, but the designs in Experiments 1 and 2 included both species primarily for replication. The sample size was too small (four planarians of each species in each experiment) to permit appropriate between-group comparisons and statistical analysis. The goal of Experiment 3 was to test whether *S. mediterranea* and *G. tigrina* were differentially affected by 10 µM of chronic xylazine.

### 5.1. Method

Eight *G. tigrina* and eight *S. mediterranea* were selected randomly from their respective colonies and housed individually in Petri dishes containing Montjuïc water. Twenty-four hours after selection, all planarians completed one negative phototaxis trial (pretest). Following this pretest, they were placed individually in Petri dishes containing a 10 µM xylazine solution. After 72 h of chronic exposure, all subjects were given another negative phototaxis trial (post-test).

### 5.2. Results and Discussion

**Figure 3:**
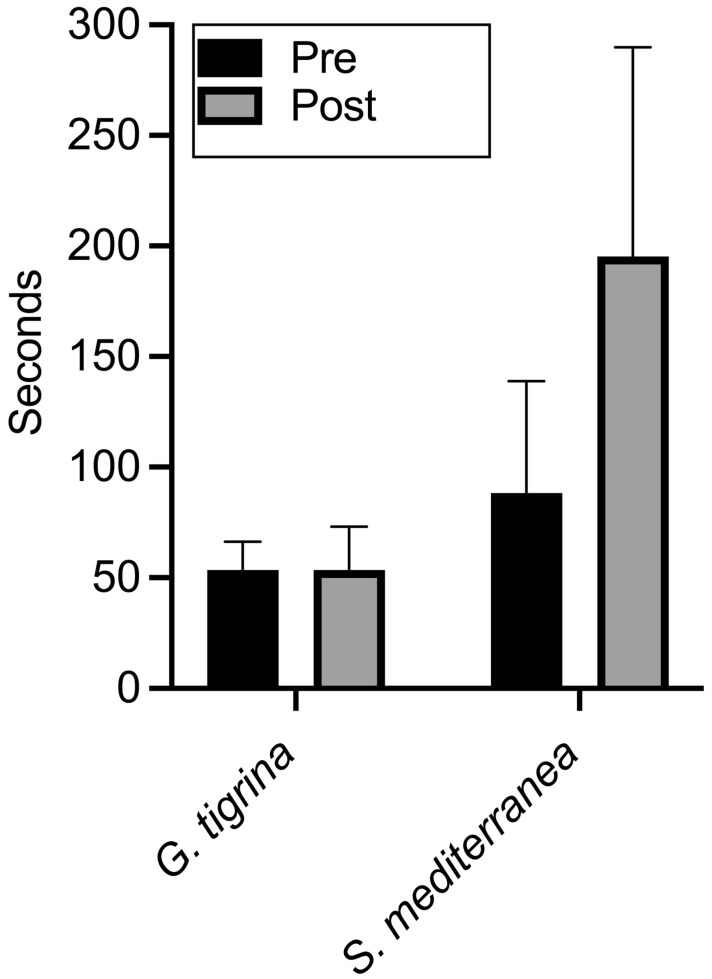
Mean pretest and post-test negative phototaxis times for *G. tigrina* (left columns) and *S. mediterranea* (right columns) in Experiment 3. Error bars represent 1 SD.

Figure 3 shows the pretest and post-test results for all planarians in both groups. All subjects crossed into the dark side of the Petri dish during the pretest, but with slower times recorded in *S. mediterranea*. For *G. tigrina*, there was no overall difference between travel times in baseline and chronic xylazine conditions. Post-test travel times were longer in three of the eight planarians. For *S. mediterranea*, travel times increased following xylazine administration in seven of the eight planarians. Although all *G. tigrina* completed the post-test within the 600 s time limit, three of the *S. mediterranea* failed to do so.

A two-way repeated-measures ANOVA showed significant effects of species (*F*(1, 14) = 22.05, *p* = 0.0003; drug, *F*(1, 14) = 7.20, *p* = 0.0178) and the interaction of species and drug (*F*(1, 14) = 7.20, *p* = 0.0178). Fisher’s LSD comparisons indicated that mean travel times between pretest and post-test differed significantly for *S. mediterranea* (*p* = 0.002, 95% CI [46.52, 167.5]). Mean travel times for *G. tigrina* did not differ significantly (*p* > 0.9999, 95% CI [−60.48, 60.48]). Pretest means between the two species were not significantly different (*p* = 0.2138, 95% CI [−91.04, 21.29]), but post-test means were significantly different (*p* < 0.0001, 95% CI [−198.0, −85.71]).

The data from Experiment 3 replicate the species differences observed in Experiments 1 and 2 by employing a group design that permitted appropriate comparisons and statistical analysis. As in the first two experiments, chronic xylazine administration did not affect negative phototaxis in *G. tigrina*, but impaired, and in some cases eliminated, negative phototaxis in *S. mediterranea*. In addition, the results from Experiment 3 further reduce the possibility that impaired phototaxis in *S. mediterranea* observed previously was due to an interaction between xylazine exposure and repeated testing, since the planarians in the between-group design were exposed to only one trial following chronic administration.

To the best of my knowledge, these data represent the first direct comparison of chronic drug effects on negative phototaxis between these two planarian species. In the present study, *S. mediterranea* were more susceptible to the effects of the one dose tested of chronic xylazine than *G. tigrina*. However, this difference is not necessarily an advantage or disadvantage when selecting an animal model for pharmacological research. If no pharmacological effects are observed, the model has limited utility. It is possible, and perhaps likely, that behavioral effects in *G. tigrina* would emerge if higher chronic doses were tested. A limitation of the present study is that only one dose of xylazine was investigated, and examination of a broader dose range may be a fruitful avenue for future research.

## 6. General Discussion

Overall, orderly results were obtained with a cost-effective planarian model, adding to a relatively small literature base regarding the behavioral effects of xylazine. Negative phototaxis proved to be stable in the absence of drug and sensitive to the chronic effects of 10 µM of xylazine when tested in *S. mediterranea*. Drug effects were evident under multiple-baseline, reversal, and group designs. Although only one dose of xylazine was tested, the methods and results provide a foundation for further investigation of a range of doses, which can be accomplished with relatively small investments in time and financial cost compared to vertebrate investigations.

Several limitations should be addressed in future endeavors. Although both the multiple-baseline and reversal designs produced convincing differences between control and drug conditions in *S. mediterranea*, a relatively small number of trials led to some interpretation difficulty, especially with a decrease in phototaxis times in some planarians that occurred toward the end of chronic exposure. Longer chronic exposure could be a solution; however, the limited number of sessions used during the current investigation reflects concern regarding the length of time planarians would go without food. Feeding planarians takes several hours, and their motility can be affected for hours afterward. Furthermore, planarians can gain considerable weight after feeding [48], and the effects of such increases on pharmacokinetics are unknown. With motility being considerably affected by chronic xylazine, at least in *S. mediterranea*, it is not clear if planarians would be able to feed during the drug phases. These questions may be assessed empirically.

Although differences in the behavioral effects of chronic xylazine between the two species tested were replicated across the three experiments, caution must be used before attributing the differences in drug effects to species alone. Although all planarians were kept in the same room under the same husbandry conditions, there could have been some confounding variables. As the two species were maintained in separate containers, it is possible that there were differences in the overall health of the two colonies. And, although attempts were made to select planarians of approximately the same length, equal length does not guarantee equal mass. In pharmacological research with vertebrate animal models, equal dosing across individual subjects is typically achieved by calculating dose as a proportion of body mass. To date, this has not traditionally been done with planarians, with investigators typically reporting the lengths of the animals. Weighing planarians is complicated but possible [48], and equalizing body mass rather than length across subjects may be a worthwhile pursuit. Further replications along with dose–response determinations are critical.

The question of generality to human affairs remains. Given that both acute and chronic xylazine affect planarian behavior, there may be potential for quick screening of pharmacological reversal agents. Drugs such as tolazoline and atipamezole are available to reverse sedation for veterinary procedures, but no drugs are approved for human use. Assessing xylazine alone and in combination with adrenergic antagonists, both established and novel, on negative phototaxis in planarians may broaden avenues for preclinical pharmacology. Given their relatively low cost compared to vertebrate models and their exemption from most IACUCs, using planarians may allow for rapid changes to research procedures, allowing for dynamic, data-based protocol development.

Finally, procedures similar to those used in the present investigation may have utility for investigating physical withdrawal from chronic xylazine administration. Withdrawal from several different psychoactive drugs has been demonstrated in planarians, and it can be evident with exposure times of less than an hour [24]. I am unaware of any prior investigations that utilized multiple-day drug exposures as described here. The reversal design used in Experiment 2 potentially allows for the assessment of withdrawal when chronic drug exposure is terminated at the end of the second phase. Adding additional trials for the purpose of recording withdrawal effects on negative phototaxis and possible hyperkinesis could be scheduled at several time points closer to the phase change to assess this possibility.

## 7. Conclusions

Chronic exposure to 10 µM xylazine impaired negative phototaxis in *S. mediterranea* but not *G. tigrina*, with effects replicated across three separate experimental designs. The data demonstrate the potential value of planarians for assessing drug effects while underscoring the need for species-specific considerations. Further work should document dose–response functions, extended exposure protocols, and evaluate the possibility of physical withdrawal symptoms.

## Data Availability

The original contributions presented in this study are included in the article. Further inquiries can be directed to the corresponding author.
